# The difference and clinical application of modified thoracolumbar fracture classification scoring system in guiding clinical treatment

**DOI:** 10.1186/s13018-023-03958-4

**Published:** 2023-07-11

**Authors:** Lu Wenjie, Zhang Jiaming, Jiang Weiyu

**Affiliations:** 1 Ningbo Sixth Hospital, Ningbo, 315000 China; 2 Zhejiang University of Traditional Chinese Medicine, Hangzhou, 310000 China

**Keywords:** Thoracolumbar fracture, Severity, Grading assessment, Clinical application

## Abstract

**Objective:**

This study aimed to evaluate the feasibility of the modified thoracolumbar injury classification and severity score system in guiding clinical treatment.

**Methods:**

A retrospective study was conducted on a cohort of 120 patients with thoracolumbar fractures who were admitted to the Department of Spinal Surgery at Ningbo Sixth Hospital between December 2019 and June 2021. The study population consisted of 68 males and 52 females, with an average age of 36.7 ± 5.7 years. The severity of the fractures was assessed based on comprehensive scores incorporating fracture morphology, neurological function, posterior ligament complex integrity, and disc injury status. The evaluation was performed using the total score T, which guided the formulation of the clinical treatment strategy. Furthermore, the study compared the treatment options, imaging data, and clinical efficacy between two classification systems.

**Results:**

The analysis of 120 patients revealed no statistically significant difference in the total score or treatment method between the TLICS system and the modified TLICS system. However, the operation rate for the modified TLICS system (73.3%) was slightly lower compared to the TLICS system (79.2%). All patients were followed up for a mean duration of 19.2 ± 4.6 months, ranging from 11 to 27 months. At the last follow-up, the visual analogue scale score was 1.94 ± 0.52, and the modified Japanese Orthopaedic Association score was 28.8 ± 4.5, indicating a significant improvement compared to the scores obtained prior to treatment. The neurological status exhibited varying degrees of improvement. Notably, the anterior vertebral height ratio was 87.10 ± 7.17%, the sagittal index was 90.35 ± 7.72%, and the Cobb angle was 3.05 ± 0.97 degrees at the last follow-up. All these measurements demonstrated statistically significant differences compared to the values observed prior to treatment (*P* < 0.05). Additionally, two cases of pedicle screw breakage and seven cases of pedicle screw wear and cutting in the vertebral body were observed at the last follow-up, resulting in varying degrees of low back pain. However, no instances of rod breakage were reported.

**Conclusion:**

The modified TLICS system is a practical tool for the classification and assessment of thoracolumbar fractures. It has guiding significance for clinical treatment, and the operation rate was slightly lower than that of TLICS system.

## Introduction

Thoracolumbar fracture is the most common type of spinal injury, accounting for approximately 90% of all spinal injuries. It is often associated with varying degrees of spinal nerve injury, which can significantly impact the patients’ quality of life. Therefore, it is crucial to rapidly and accurately assess the fracture injury status and employ appropriate treatment measures to achieve better therapeutic outcomes [1]. A reliable and effective thoracolumbar fracture classification scoring system is essential to achieve this objective [2–4]. Currently, the most commonly used classification systems for thoracolumbar fractures in clinical practice include Denis classification, AO classification, and TLICS classification. However, each system has its own limitations [5]. For instance, Denis classification is too simplistic and does not cover all fracture types, while AO classification is too complex, which can pose challenges in clinical application [6, 7]. Although the TLICS classification was a step forward in combining PLC integrity, neurological function, and injury state of injured vertebrae, some scholars have raised questions about the assignment of scores in each subcategory [8]. Therefore, to improve the TLICS classification, the author added the “intervertebral disc injury state” subcategory, reduced the score of the “PLC integrity” subcategory, and proposed a modified TLICS classification system. The feasibility of guiding clinical treatment using this modified TLICS classification system was investigated by analysing the clinical data of 120 patients with thoracolumbar fractures admitted to the Department of Spinal Surgery at Ningbo Sixth Hospital from December 2019 to June 2021. The study aimed to provide valuable insights for the clinical diagnosis and treatment of thoracolumbar fractures.

## Patients and methods

### Patients

The inclusion criteria for this study consisted of patients diagnosed with fresh, single-segment thoracolumbar fractures within 1 week after fracture, without combined multiple severe trauma and medical diseases, having complete clinical and imaging data (including preoperative thoracolumbar frontal and lateral radiographs, CT + 3D reconstruction, and MRI), and providing informed consent signed by the patient and their family members. Patients with multisegmental or old thoracolumbar fractures and severe multiple trauma such as concomitant craniocerebral injury, osteoporotic fractures, incomplete imaging data, or lost visits were excluded from the study.

General Information: Prior to commencing the study, all participants received training on the modified TLICS system. Subsequently, participants were selected based on inclusion and exclusion criteria, and the purpose and significance of the survey were declared. After obtaining informed consent from the patients, a total of 131 individuals with thoracolumbar fractures were initially included in the study. However, nine cases were excluded due to failure to adhere to timely follow-up after discharge, and two cases were excluded due to re-fracture within 3 months following discharge. As a result, the final sample size consisted of 120 patients, comprising 68 males and 52 females, with ages ranging from 22 to 65 years (mean age: 36.7 ± 5.7 years). The distribution of fractures by segment was as follows: T11—14 cases, T12—45 cases, L1—54 cases, and L2—seven cases. The Frankel classification was used to assess spinal nerve function, with the distribution as follows: Grade A—three cases, Grade B—eight cases, Grade C—12 cases, Grade D—28 cases, and Grade E—69 cases. The causes of injury included 63 cases of traffic accidents, 27 cases of crushing injuries, 18 cases of falls, and 12 cases of other injuries. The study protocol has received approval from the Medical Ethics Committee of Ningbo Sixth Hospital, and all patient-related data used in this study have been authorized for use and publication by the patients themselves or their legal guardians.

## Methods

**Fig. 1:**
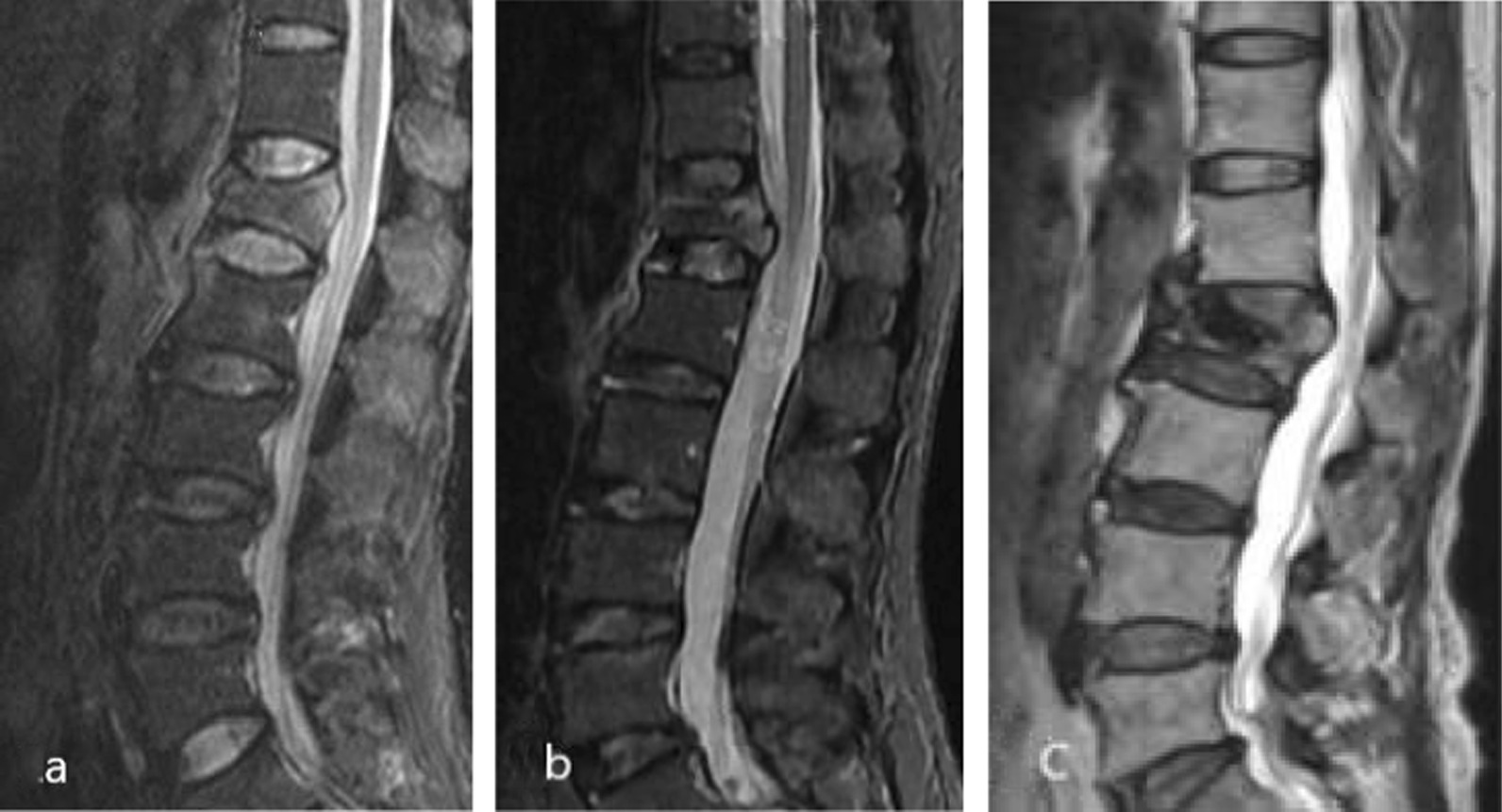
Pre-treatment thoracolumbar MRI. According to the imaging features of intervertebral disc injury, it was divided into three categories: no injury, mild injury, and moderate-to-severe injury. **a** Intervertebral disc was not damaged (0 point); **b **mild injury of intervertebral disc, signal change, no endplate injury, and with or without gap change (1 point); and **c **intervertebral disc moderate-to-severe injury, signal changes, endplate rupture, intervertebral disc content hernia into the vertebral body, and intervertebral space change (2 points)

Based on clinical data and literature reports, this study proposed a modified TLICS classification scoring system [9]. The changes include reducing the score assigned to the “PLC integrity” subclass and increasing the “Intervertebral disc injury status” subclass. The “PLC integrity” subclass scores 0, 1, and 2 for no damage, suspicious damage, and damage, respectively. The “intervertebral disc injury state” subclass evaluation refers to the Sander classification and is divided into no injury, mild injury, and moderate-to-severe injury, scoring 0, 1, and 2 points, respectively [10] (Fig. 1). The subclasses of fracture morphology and neurological function are the same as those of the TLICS system. The severity of the fracture is evaluated based on the total score T of the four subcategories, and clinical treatment is guided accordingly (Table 1). Conservative treatment, such as bed rest, waist pillow, brace treatment, and TCM conditioning, is recommended when T ≤ 3 points. Conservative or surgical treatment is adopted when T = 4, based on the patient’s vital signs and quality of life requirements. Surgical treatment is performed when T ≥ 5, using techniques such as vertebroplasty, kyphoplasty, or pedicle screw systems.

**Table 1:** Modified TLICS staging scoring system

Subcategory	Score
*Fracture morphology*	
Compressive	1
Bursting	2
Reduced force and rotational	3
Distraction	4
*Neurological function state*	
No injury	0
Nerve root injury	2
Complete spinal cord/Conus injury	2
Incomplete spinal cord/Conus injury	3
Cauda equina injury	3
*PLC integrity*	
No damage	0
Suspicious damage	1
Damage	2
*Intervertebral disc injury state*	
No damage	0
Mild injury	1
Moderate and severe injury	2

### Clinical efficacy and imaging materials

The following parameters were assessed and recorded at three time points: before treatment, 1 month after treatment, and at the last follow-up: VAS score, JOA score, local Cobb angle, sagittal index, anterior vertebral height, and Frankel grade of spinal nerve function. Moreover, occurrences of internal fixation failure, other vertebral fractures, symptomatic kyphosis, and any other complications were meticulously documented.

### Data statistics

The imaging materials were measured using Photoshop CS2 v9.0 software, and the collected data were processed and analysed using SPSS 25.0 software. Count data were presented as number/percentage and analysed using the Chi-square test. Measurement data were expressed as mean ± standard deviation, and group t-tests were utilized for comparisons between the two groups. A significance level of *P* < 0.05 was employed to determine statistical significance.

## Results

### General situation

According to the TLICS system, among the 120 patients, 19 patients scored T ≤ 3 points, 26 patients scored T = 4 points, and 75 patients scored T ≥ 5 points. Based on this system, 25 patients were recommended for conservative treatment, while 95 patients were recommended for surgical treatment. On the other hand, utilizing the modified TLICS system, out of the same 120 patients, 23 patients scored T ≤ 3 points, 26 patients scored T = 4 points, and 71 patients scored T ≥ 5 points. Using the modified system, 32 patients were recommended for conservative treatment, while 88 patients were recommended for surgical treatment. The Chi-square test revealed no significant difference in the total score T and the choice of treatment methods between the two systems (*P* = 0.782 and *P* = 0.288). However, the proportion of patients undergoing surgery was slightly lower for those assessed with the modified TLICS system (73.3%) compared to those assessed with the TLICS system (79.2%) (Table 2).

**Table 2:** Comparison of TLICS system and modified TLICS system total score T and treatment methods

Total score and treatment/system		TLICS system	Modified TLICS system	Statistic	*P*-value
	≤ 3 points	19	23		
Total score	= 4 points	26	26	*x*^2^ = 0.491	0.782
	≥ 5 points	75	71		
Treatment	Conservative treatment	25	32	*x*^2^ = 1.127	0.288
	Surgical treatment	95	88		

*P* < 0.05 indicates that the difference is statistically significant

**Fig. 2:**
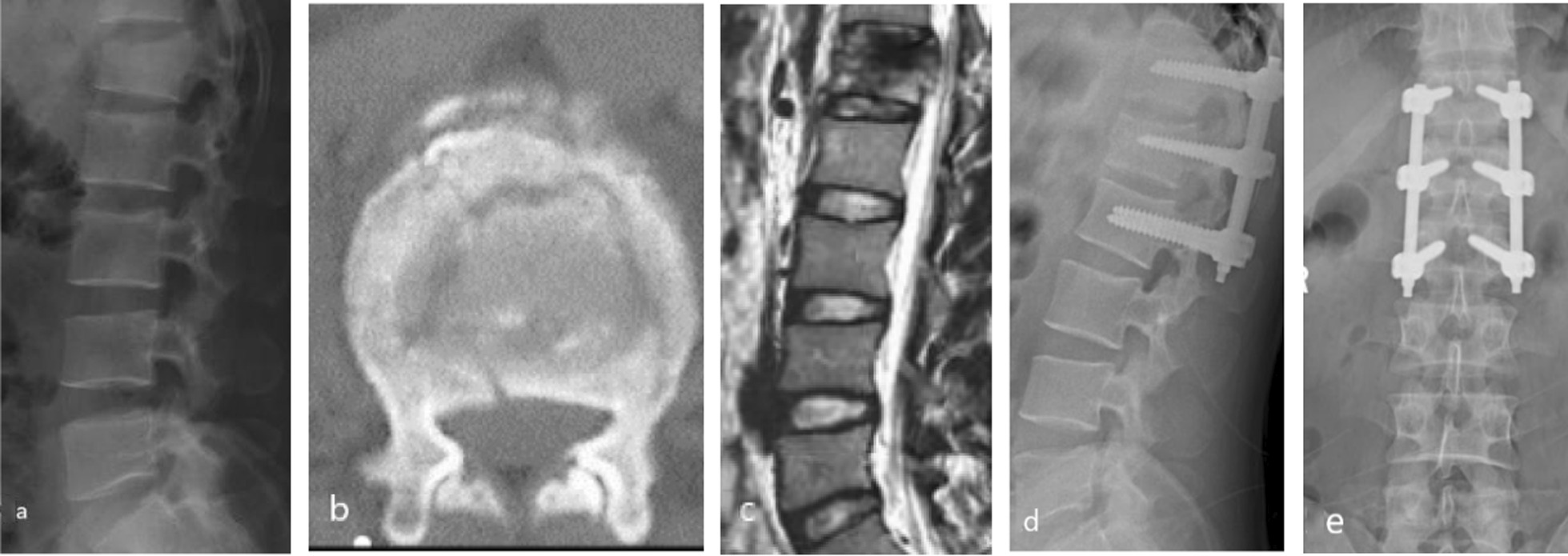
The patient was a 46-year-old man with low back pain caused by trauma for 1 day, **a**, **b** lumbar 1 vertebral burst fracture; **c **lumbar 1 burst fracture, no nerve function damage, PLC suspicious injury, and severe disc injury. The modified TLICS system: burst fracture (2 points), PLC suspicious injury (1 point), severe intervertebral disc injury (2 points), and no nerve injury (0 point), total score 5 points, should be treated surgically. The TLICS system: burst fracture (2 points), PLC suspicious injury (2 points), and no nerve injury (0 point), total score 4 points, conservative or surgical treatment, there is a difference between the two. According to the modified TLICS system for surgical treatment, **d, e** after 1 month, the lumbar spine sequence recovered well, and no kyphosis occurred

**Fig. 3:**
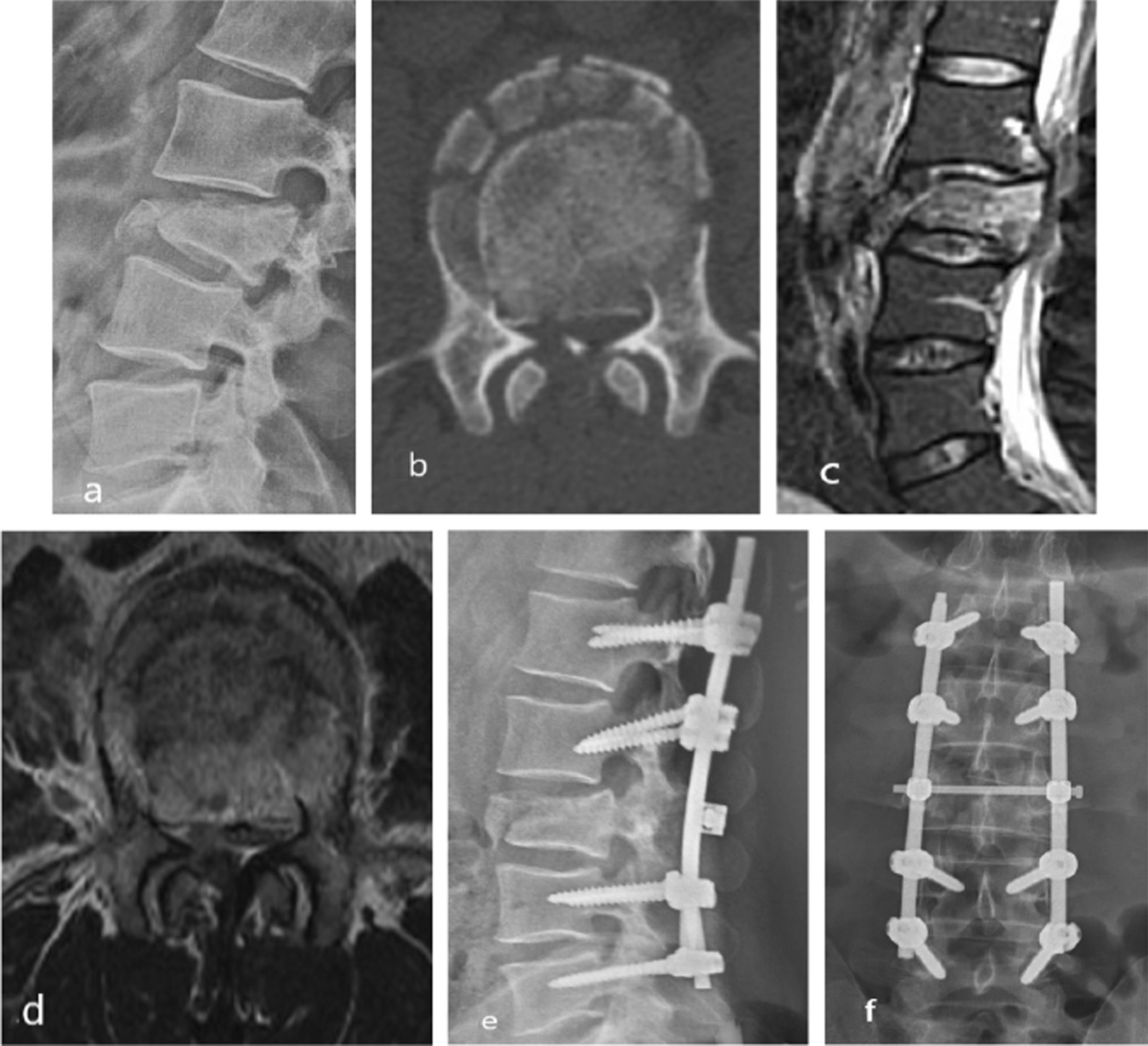
A 32-year-old man presented with low back pain and right lower limb numbness for 2 days. Imaging showed a lumbar 3 burst fracture (**a**, **b**) and a fresh burst fracture of the lumbar 3 vertebral with spinal stenosis and cauda equina compression (**c**, **d**). The modified TLICS system was used, which revealed a total score of 8 points, indicating that surgical treatment was necessary. The TLICS system also yielded a total score of 8 points, confirming the need for surgical intervention. The patient underwent surgery and, as evidenced by imaging at 1 month post-operation (**e**, **f**), had a good recovery of lumbar function without kyphosis

Of the patients who underwent conservative treatment, four later required surgical intervention due to worsening kyphosis of the injured vertebra and delayed nerve injury. Ultimately, 28 patients were treated conservatively while 92 underwent surgery. All patients were followed up for an average of 19.2 ± 4.6 months after discharge. At the last follow-up, 11 patients reported persistent low back discomfort after surgery, which was managed with symptomatic treatment such as anti-inflammatory and analgesic medication, resulting in a decrease in VAS score to 2.1 points. In addition, two patients experienced broken pedicle screws, while no rods were broken. Seven patients had pedicle screws that showed varying degrees of wear and cutting in the vertebral body, which was associated with varying degrees of back pain (Figs. 2 and 3).

### Typical cases

#### Imaging materials

Compared to the pre-treatment measurements, the Cobb angle, sagittal index, and anterior vertebral height showed significant improvements at the last follow-up. Statistical analysis using the *t*-test demonstrated a significant difference (*P* < 0.001) (Table 3).

**Table 3:** Comparison of imaging data before treatment and at the last follow-up (*x* ± *s*)

Indicators/time	Before treatment	At the last follow-up	Statistic	*P*-value
Anterior vertebral height (%)	58.25 ± 11.37	87.10 ± 7.17	*t* = − 23.511	< 0.001
Sagittal index (%)	60.11 ± 7.28	90.35 ± 7.72	*t* = − 31.218	< 0.001
Cobb angle (°)	25.02 ± 3.91	3.05 ± 0.97	*t* = 59.741	< 0.001

*P* < 0.05 indicates that the difference is statistically significant

### Clinical indicators

At the last follow-up, the VAS score and modified JOA score were recorded as 1.94 ± 0.52 and 28.8 ± 4.5, respectively. These scores exhibited significant differences compared to the pre-treatment values of 7.91 ± 0.83 and 16.8 ± 5.1, respectively. Based on the Frankel classification of spinal nerve function, there were four cases graded as A, two cases graded as B, six cases graded as C, 16 cases graded as D, and 92 cases graded as E. Statistical analysis using the Chi-square test indicated a significant difference when compared to the preoperative values (*P* < 0.05). Importantly, no cases of worsened nerve injury or nonunion of fractures were observed (Table 4).

**Table 4:** Clinical indicators before treatment and at the last follow-up (n)

Clinical indicators/time		Before treatment	At the last follow-up	Statistic	*P*-value
VAS score		7.91 ± 0.83	1.94 ± 0.52	*t* = 66.771	< 0.001
Modified JOA score		16.8 ± 5.1	28.8 ± 4.5	*t* = − 19.327	< 0.001
	Grade A	4	4		
	Grade B	6	2		
Frankel classification	Grade C	16	6	*x*^2^ = 14.944	0.005
	Grade D	29	16		
	Grade E	65	92		

*P* < 0.05 indicates that the difference is statistically significant, and *P* < 0.001 indicates that the difference is statistically significant

## Discussion

The thoracolumbar spine (T11-L2) serves as a transitional region between the relatively immobile thoracic spine and the more mobile lumbar spine. It exhibits a gradual transition of the facet joint surface from the coronal plane of the thoracic spine to the sagittal plane of the lumbar spine. Owing to its unique anatomical structure and stress distribution, the thoracolumbar spine is prone to injury. Such injuries often involve the spinal cord, conus medullaris, and cauda equina, resulting in a high disability rate that significantly impacts patients’ daily life and work capacity. Consequently, the clinical management of thoracolumbar fractures holds paramount importance [11, 12]. It is imperative to establish a standardized and practical scoring classification system that considers the mechanism of injury, aids in prognosis assessment, and guides the selection of appropriate surgical interventions. However, existing classification methods fail to meet these aforementioned requirements.

The modified TLICS system has been developed based on the TLICS system. It primarily assesses the injury status and severity of thoracolumbar fractures by considering the fracture morphology, neurological function, PLC integrity, and adjacent intervertebral disc status [13, 14]. The evaluation of fracture morphology focuses on determining the immediate stability of the spine, while the assessment of intervertebral disc injury and PLC integrity aims to evaluate the long-term stability of the spine. Additionally, the neurological status is assessed to determine the neurological stability. Compared to the TLICS system, the modified TLICS system introduces several changes. It reduces the weightage assigned to the “PLC integrity” subcategory and increases the emphasis on evaluating the “disc injury state” subcategory. This refinement allows for a more detailed assessment of the roles played by the three columns in maintaining spinal stability. Furthermore, it recognizes the significance of the anterior and middle columns in preserving the biomechanics and long-term stability of the spine. Notably, there are no differences in the evaluation of fracture morphology and neurological function between the original system and the modified TLICS system.

### The subcategory assignment of “PLC integrity” is reduced

The “PLC integrity” subcategory score is reduced in the modified TLICS system due to several reasons. First, clinicians may overemphasize the impact of PLC integrity on spinal stability. The purpose of treating thoracolumbar fractures is to reconstruct the spinal sequence and restore stability. However, the role of the three columns in spinal stability remains unclear, with different scholars having different or even contradictory understandings. For example, Izzo et al. [15] believed that the anterior and middle columns of the spine are more important in maintaining axial mechanical stability and bear about 70–80% of the axial compressive stress of the spine. Yu et al. [16] argued that the PLC bears more than 60% of the tension load when the spine is subjected to flexion deformity stress, and is essential for maintaining stability. The consistency in this area is not high and is more subjective. For example, Hartmann et al. [17], in a retrospective study, found that the sensitivity and specificity of X-ray and CT bone parameters in detecting PLC injury are not high. Rihn et al. [18] believed that the high signal of PLC in MRI fat suppression images indicated damage, and although the sensitivity was high, the specificity was only 68.4%. Therefore, the modified TLICS system reduces the score system of PLC integrity to improve the objectivity of the TLICS total score.

### Increased “disc injury status” subcategory assessment

When a significant force impacts the human body, some of the liquid biomechanical properties in the intervertebral disc manifest as solid biomechanical properties. Thus, severe thoracolumbar fractures often result in intervertebral disc injury, particularly in the adjacent vertebrae [19]. Intervertebral discs have poor blood supply, making recovery difficult, and scar tissue formation often replaces injured tissue, which affects long-term spinal stability [20, 21]. Despite the importance of the injured intervertebral disc in the spinal passive stabilization system, clinical attention has not been paid to this issue, contributing to delayed kyphosis after severe thoracolumbar fracture surgery [22]. Mi et al. [23] found that postoperative kyphosis in 84 patients with thoracolumbar fractures resulted mostly from the loss of upper intervertebral disc height in the injured vertebra. Similarly, Hou et al. [24] showed that intervertebral disc injury was the primary cause of kyphosis progression after thoracolumbar fracture and kyphosis recurrence after posterior reduction and fixation. Thus, assessing the “disc injury status” is crucial to accurately evaluate the long-term stability of the spine.

In this study, no significant differences were found in the total score and treatment choices between the modified TLICS system and the TLICS system in the cohort of 120 patients with thoracolumbar fractures. However, the modified TLICS system resulted in a slightly lower rate of surgical intervention compared to the TLICS system. Ultimately, based on the guidance of the modified TLICS system, conservative treatment was administered to 28 cases while surgical treatment was administered to 92 cases. At the last follow-up, significant improvements were observed in various outcome measures. The VAS score, modified JOA score, anterior vertebral height ratio, sagittal index, and Cobb angle demonstrated statistically significant differences when compared to the pre-treatment values. Moreover, neurological function exhibited variable degrees of improvement (*P* < 0.05).

### Limitations

This study has several limitations that should be acknowledged. Firstly, the economic status of patients and other relevant factors were not considered when determining the treatment approach. Patients may have chosen treatment methods that were not aligned with the improved TLICS system due to their individual financial circumstances. This could potentially introduce bias in the results, especially for patients with a total score of T = 4, leading to an increase in the non-surgical treatment group. Secondly, the assignment of scores for each subclass of the classification system requires more scientific and reasonable definitions, which should be supported by biomechanical principles and extensive clinical studies. Further refinement and validation of the scoring criteria are necessary to enhance the accuracy and reliability of the classification system. Lastly, this study was conducted at a single centre with a small sample size and short-term follow-up. Therefore, the generalizability of the findings may be limited. Future research should aim for multi-centre collaboration, larger sample sizes, and long-term prospective studies to provide more robust evidence.

## Conclusion

In conclusion, although the modified TLICS system fails to address the root defects of the original typing system, it can mitigate the defects’ impact by increasing or decreasing the assignment of each subclass. Consequently, the modified TLICS system is currently a more reasonable and effective treatment method. This system has significant guidance for formulating treatment strategies for patients with thoracolumbar fractures and has considerable value for promotion.

## Data Availability

The datasets used and/or analysed during the current study available from the corresponding author on reasonable request.
